# A Hybrid Model of In-Person and Telemedicine Diabetes Education and Care for Management of Patients with Uncontrolled Type 2 Diabetes Mellitus: Findings and Implications from a Multicenter Prospective Study

**DOI:** 10.1089/tmr.2024.0003

**Published:** 2024-02-19

**Authors:** Ayla M. Tourkmani, Turki J. Alharbi, Abdulaziz M. Bin Rsheed, Azzam F. Alotaibi, Mohammed S. Aleissa, Sultan Alotaibi, Amal S. Almutairi, Jancy Thomson, Ahlam S. Alshahrani, Hadil S. Alroyli, Hend M. Almutairi, Mashael A. Aladwani, Eman R. Alsheheri, Hyfaa Salaheldin Sati, Budur Aljuaid, Abdulaziz S. Algarzai, Abood Alabood, Reuof A. Bushnag, Wala Ghabban, Muhammed Albaik, Salah Aldahan, Dalia Redda, Ahmed Almalki, Noura Almousa, Mohammed Aljehani, Alian A. Alrasheedy

**Affiliations:** ^1^ Family and Community Medicine Department, Chronic Illness Center, Prince Sultan Military Medical City, Riyadh, Saudi Arabia.; ^2^ Health Services, Ministry of Defense, Riyadh, Saudi Arabia.; ^3^ Department of Pharmacy Practice, College of Pharmacy, Qassim University, Qassim, Saudi Arabia.

**Keywords:** diabetes mellitus, telehealth, hyperglycemia, uncontrolled diabetes, therapeutic inertia

## Abstract

**Background:**

Patients with uncontrolled type 2 diabetes mellitus (T2DM) require close follow-up, support, and education to achieve glycemic control, especially during the initiation or intensification of insulin therapy and self-care management. This study aimed to describe and evaluate the impact of implementing a hybrid model of in-person and telemedicine care and education on glycemic control for patients with uncontrolled T2DM (hemoglobin A1c [HbA1c] ≥9%) during the coronavirus disease pandemic.

**Methods:**

This prospective multicenter-cohort pre-/post-intervention study was conducted on patients with uncontrolled T2DM. This study included three chronic illness centers affiliated with the Family and Community Medicine Department at Prince Sultan Military Medical City in Riyadh, Saudi Arabia. A hybrid model of in-person (onsite) and telemedicine care and education was developed. This involved implementing initial in-person care at the physicians' clinic and initial in-person education at the diabetes education clinic, followed by telemedicine services of tele-follow-ups, support, and education for an average 4-month follow-up period.

**Results:**

Of the enrolled 181 patients, more than half of the participants were women (*n* = 103, 56.9%). The mean age of participants (standard deviation) was 58.64 ± 11.23 years and the mean duration of diabetes mellitus was 13.80 ± 8.55 years. The majority of the patients (*n* = 144; 79.6%) were on insulin therapy. Overall, in all three centers, the hybrid model had significantly reduced HbA1c from 10.47 ± 1.23% to 7.87 ± 1.59% (mean difference of reduction 2.59% [95% confidence interval (CI) = 2.34–2.85%], *p* < 0.001). At the level of each center, HbA1c was reduced significantly with mean differences of 3.17% (95% CI = 2.81–3.53%), 2.49% (95% CI = 1.92–3.06%), and 2.16% (95% CI = 1.76–2.57%) at centers A, B, and C, respectively (all *p* < 0.001).

**Conclusion:**

The findings showed that the hybrid model of in-person and telemedicine care and education effectively managed uncontrolled T2DM. Consequently, the role of telemedicine in diabetes management could be further expanded as part of routine diabetes care in primary settings to achieve better glycemic control and minimize nonessential in-person visits when appropriate.

## Introduction

Type 2 diabetes mellitus (T2DM) is a chronic metabolic disease characterized by hyperglycemia, which accounts for ∼90% of all diabetes mellitus cases.^1–4^ Persistent hyperglycemia in patients with uncontrolled T2DM is associated with many complications, including impaired pancreatic β-cell function (glucotoxicity), microvascular complications (nephropathy, neuropathy, and retinopathy), and macrovascular complications (coronary heart disease, peripheral artery disease, and cerebrovascular diseases). Consequently, this leads to higher morbidity and mortality rates.^1,3,5–9^ Globally, the prevalence of T2DM has substantially increased in recent decades.^2,10,11^ It increased from ∼108 million in 1980 to 536.6 million adults with T2DM in 2021, and this is projected to further increase to reach 783.2 million by 2045.^12,13^

Many pharmacological therapies with positive health outcomes are currently available for the management of T2DM.^6,14–16^ However, as T2DM is directly associated with lifestyle, nutrition, dietary intake, and physical activity, effective diabetes management involves both nonpharmacological and lifestyle interventions. These are essential for achieving better glycemic control and providing sustainable therapeutic outcomes.^14,16–20^ Therefore, it is crucial to empower patients with the knowledge and skills to manage their disease appropriately using antidiabetic medications/insulin therapy and appropriate lifestyle changes (self-care management).^20–23^ Hence, effective patient education and training to increase awareness and self-efficacy in management of T2DM cannot be undermined.^17,21–24^

Patients with poorly controlled diabetes mellitus (HbA1c ≥9%) are at a higher risk for complications and mortality.^25,26^ A recent study showed that the excess mortality associated with T2DM was 25–45% higher among patients with controlled T2DM (HbA1c <7%) than in individuals without diabetes and cardiovascular disease, and that the risk was substantially higher (82–151%) in patients with HbA1c ≥9%.^26^ Consequently, to reduce the risk for premature mortality, patients with uncontrolled diabetes require intensification of therapy and close monitoring and guidance, including the initiation or intensification of insulin therapy when clinically required to achieve glycemic control.^9,27,28^

However, one of the major barriers to achieving this is the delay in treatment intensification, despite suboptimal glycemic control, known as “therapeutic inertia.”^27,28^ Therapeutic inertia in diabetes refers to the lack of timely modification/adjustment of the treatment plan, despite high HbA1c levels.^29^ This prolongs the duration of hyperglycemia, which subsequently poses an increased risk for diabetes-related complications and mortality.^27^

Several factors contribute to the development of therapeutic inertia, resulting in failure to achieve the therapeutic targets, including initiation of insulin therapy when clinically required. These factors may be related to health care providers, patients, and systems.^9,27,28^ The factors related to health care providers include lack of updated knowledge, time constraints (limited consultation time), lack of support staff to train patients on the injection site and glucose monitoring, and fear of hypoglycemia for initiation or intensification of insulin therapy. The factors related to patients include low health literacy, low self-efficacy, lack of education and support, fear of injections, and nonadherence to appointments.

The factors related to the health care system include high workload, limited follow-ups, and lack of access to education and training for diabetes self-management.^9,27,28^ Therefore, one of the solutions to address these barriers, especially physicians' limited time, inadequate follow-up appointments to monitor and/or adjust the therapy, inadequate training of patients, patients' concerns, and the burden of frequent in-person visits, is to introduce initiatives such as patient education and support (such as through certified diabetes educators [CDEs]) utilizing telemedicine as part of primary care.^30–33^ This could be implemented in a hybrid model of in-person (on-site) and tele-follow-up to provide education, close monitoring, and individualized guidance to improve the glycemic level in high-risk, poorly controlled diabetes patients who require guidance for the initiation and titration of insulin therapy.

Diabetes educators play a vital role in helping patients manage their diabetes and achieve glycemic control.^34^ They initially assess patients' knowledge of self-management, patients' knowledge of the disease (such as on diabetes, hyperglycemia, and hypoglycemia), medications, and the impact of lifestyle behaviors on diabetes. Consequently, they provide diabetes education, including clinical information on insulin and injectable medications, such as dose, injection site and technique, checking the expiry date of injectable medication before use, appropriate use of the glucometer, and self–monitoring of blood glucose (SMBG) (such as skills and self-efficacy).

In addition, they address health literacy through diabetes education materials (such as monitoring blood glucose, high fluid intake, healthy diet plans, sports activities and exercises, and how to perform and break fasts during Ramadan).^34–36^ Moreover, they could help patients using technology (telemedicine) to provide thorough and timely clinical diabetes care based on the therapeutic plan approved by the treating physicians (such as insulin titration and dosage adjustment based on glycemic targets).

During the coronavirus disease (COVID-19) pandemic, the use of telemedicine in various forms has witnessed an exponential increase in the delivery of health care services.^37,38^ Several technologies and applications have been used, such as virtual reality, video conferencing, telephonic consultations, virtual visits, telemonitoring, and tele-follow-ups.^38^

The rapid adoption of telemedicine occurred because the COVID-19 pandemic had substantially interrupted the routine management of patients with diabetes mellitus, especially in the early phases of the pandemic due to precautionary measures, such as lockdowns, cancellation of in-person appointments, and patients' fear of being infected with COVID-19 virus when attending clinics and hospitals.^39–43^ Building on the experience gained during the pandemic, telemedicine and teleconsultations are increasingly being used to deliver health care for patients with diabetes. This is particularly important for improving access to health care, to positively influence self-care and self-management behaviors, and to achieve better health outcomes through tele-follow-up and tele-education.

A study by Mishra et al. in 2021 showed that diabetes education, including insulin injection technique with telemedicine, was feasible and effective in the management of diabetes patients and was well received by 96% of COVID-19 patients with diabetes mellitus.^44^ However, most of the studies conducted during the COVID-19 pandemic reported experiences with the rapid shift to telemedicine as an alternative to in-person visits for managing T2DM during the lockdown period (i.e., a telemedicine-only model of care).^45–51^ Therefore, there is a paucity of data on the effectiveness of tele-follow-up and individualized tele-education (through virtual diabetes education clinics) incorporated as part of primary care for the management of patients with high-risk T2DM. This involves implementing initial in-person (onsite) care and education, followed by telemedicine care and education (hybrid model).

Therefore, this study aimed to describe and evaluate the impact of implementing a hybrid model of in-person and telemedicine care and education in three chronic illness centers (CICs), affiliated with a Family and Community Medicine Department, on the glycemic control for patients with poorly controlled diabetes mellitus (HbA1c ≥9%) during COVID-19 pandemic. We believe this study would provide new insights into the role of a hybrid model of care in the management of patients with T2DM.

## Methods

### Study design, setting, and population

A prospective multicenter-cohort pre-/post-intervention study was conducted on patients with uncontrolled T2DM (HbA1c level ≥9%). This study included three peripheral CICs (Yasmine Center [A], Almanar Center [B], and South Riyadh Center [C]) affiliated with the main CIC center of the Family and Community Medicine Department at Prince Sultan Military Medical City in Riyadh, Saudi Arabia. The centers are located at different geographic locations in Riyadh city (north, east, and south). The study was conducted between March and September, 2021.

### Diabetes care in the virtual diabetes educator clinic

A virtual diabetes educator clinic was established in the main center to serve all patients in the peripheral centers. The structure, workflow, procedures, and scheduling of the virtual clinic were discussed and agreed upon among the clinical teams of the centers (i.e., physicians, clinical pharmacist, and diabetes educators) and the department administration to ensure the smooth running of in-person education and virtual clinic.

In addition, to standardize diabetes education, a protocol, including educational materials on diabetes, guidance on basal insulin titration, and home blood glucose monitoring, was established. The aim of the virtual diabetes education clinic was to efficiently serve all patients in the three centers, improve glycemic control during the COVID-19 pandemic and beyond, ensure proper insulin and other injectable medication titration using the established protocol of the main center for all high-risk T2DM patients in the peripheral centers, and empower the patients with appropriate and tailored educational guidance and support. Consequently, a virtual clinic was established as an alternative to in-person diabetes education follow-up visits to reduce the potential risk for exposure to COVID-19, especially in high-risk patients with uncontrolled T2DM.

**FIG. 1.**
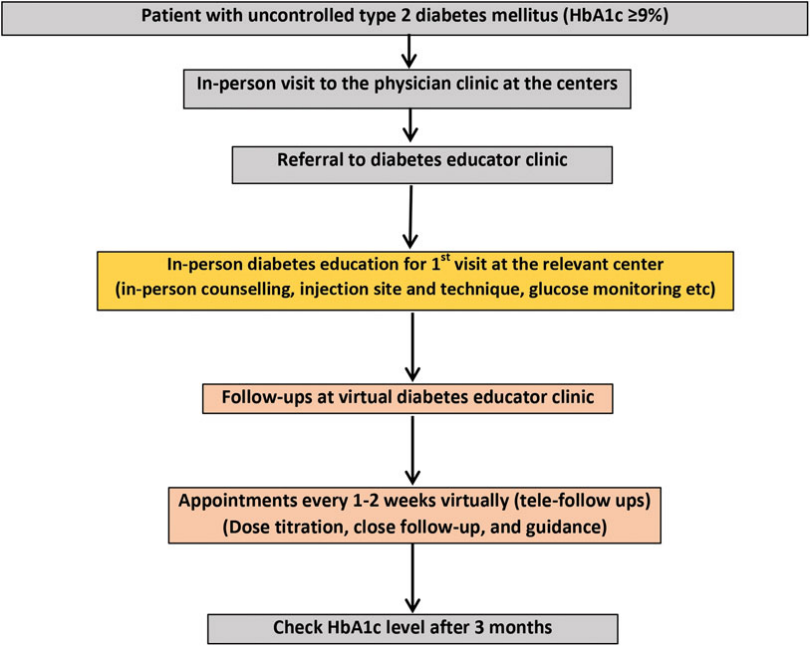
The hybrid model of in-person and telemedicine care and education.

The virtual clinic education service was centralized and located at the main center. For the in-person education clinic, there is one clinic in each peripheral center that operates for only one full working day every week. The virtual and in-person clinics were run by five full-time nurses with clinical experience in diabetes education and were CDEs. In this hybrid model of in-person care and telemedicine, T2DM patients with uncontrolled glycemic level (HbA1c ≥9%) were enrolled in the virtual diabetes educator clinic for education, diabetes care, and follow-up with more focus on patients who were prescribed injectable medications (insulins or other antidiabetic injectable medications). The first visit was scheduled as an in-person care visit at the relevant center and subsequent follow-up visits were performed through the centralized virtual clinic whenever appropriate and applicable (Fig. 1).

In this hybrid model of care, all patients were initially seen by physicians at the three centers, and their diabetes care and appropriate therapeutic plans were determined, documented, and entered electronically into the patient records in the electronic system. Treating physicians referred the patients to the diabetes education clinic using a virtual clinic-designated form. The form included all required patient information (name of the center, date of referral, patient's name, patient's medical record number, patient's contact numbers, recent HbA1c value, and drug regimens prescribed by the treating physician in the center).

The treating physician's responsibilities were to prescribe glucometers, lancets, accessories, and antidiabetic medications, including injectable medications, in appropriate quantities to cover the titration period of medications during the follow-up period with diabetes educators with a clear written plan. Consequently, as mentioned earlier, the first visit to the diabetes educator recommended delivery through an in-person care clinic at the relevant center. This is due to the need for proper education and training to ensure that patients receive the appropriate information regarding their medications (dose and frequency), glucometer use, injection technique, especially for patients who were newly started on insulin and injectable medications, and lifestyle modifications (diet and exercise). Second, subsequent follow-up with diabetes educators was recommended to be undertaken virtually, utilizing phone calls and the WhatsApp application.

Telecare and education were provided through phone calls to have an interactive, real-time intervention. Moreover, WhatsApp application was used to provide additional written instructions, educational materials, and audio-visual aids. In addition, the patients were provided with written educational materials in print and digital format regarding insulin injection techniques, hypoglycemia, diabetes, physical exercises, Ramadan fasting and diabetes, and a healthy diet for patients with diabetes. Short Message Service was used to send reminders to the patients about their scheduled appointments with the virtual clinic.

During the virtual sessions, the diabetes educators discussed and reviewed with the patients or their caregivers on the SMBG readings (such as SMBG readings before lunch and 2 h after lunch). In case of any difficulty in virtually reviewing the SMBG readings, the patients were asked to send them using the home blood glucose monitoring form through WhatsApp for further confirmation and guidance. Consequently, based on the findings during the virtual consultation, the diabetes educator guided the patients toward proper titration of insulin and other injectable medications through the agreed protocols and plans at the centers.

In addition to insulin titration and dosage adjustments, diabetes educators provided tailored and individualized diabetes education and suggestions during the virtual session if the patient experienced any hypoglycemic or hyperglycemic events. In addition, during the virtual sessions, diabetes educators encouraged the patients to ask if they had any question or need related to diabetes care and responded to all patient questions. Appropriate educational materials were sent through WhatsApp for further guidance and education relevant to the patients' needs, as discussed during the session.

The frequency of virtual appointments was every 1–2 weeks for close follow-up and review of the agreed plan, including insulin titration, by a diabetes educator. As part of the clinical protocol, diabetes educators work closely with consultant physicians and the project manager (clinical pharmacist) for any further intervention or therapeutic plan change to redesign the plan based on SMBG, patient preferences, or patient condition during follow-up (such as any special dose titration or recurrent hypoglycemia episode). All interventions (insulin titration and dose adjustment, addition, or discontinuation) and any change in the oral medications during the follow-up period when attending the virtual diabetes educator clinic were recorded. The average follow-up period was 4 months.

All patients enrolled in the service should be checked for HbA1c at their relevant center 3 months after pre-enrollment HbA1c. Once the patient reached the agreed target glycemic level planned by the treating physician, the patient was discharged from the diabetes educator service and resumed usual care with normal appointments with the treating physician.

### Sample size and inclusion and exclusion criteria

The inclusion criteria were patients with T2DM, patients >18 years of age, patients with a recent HbA1c value ≥9% (pre-enrollment), and patients with a post-enrollment HbA1C value. Consequently, all patients with HbA1c values <9% at baseline, patients with no HbA1c values after attending the virtual diabetes educator clinic, or those who opted not to participate in the study were excluded. In addition, patients or their caregivers were required to have smartphones with internet connectivity to be able to communicate with the diabetes educators and receive digital content and instructions.

All patients who met the inclusion criteria during the study period and consented to participate were enrolled. A total of 181 patients were enrolled in this study.

### Data management and analysis

Descriptive analyses were performed, including mean and standard deviation (SD), median, and interquartile range (Q1–Q3) for continuous variables, and frequency and percentage for categorical variables. Inferential statistics, including paired *t*-test, were used to report changes in pre- and post-intervention HbA1c levels. One-way analysis of variance with *post hoc* analysis were used to determine the differences in HBA1c among independent groups. Statistical significance was set at *p* < 0.05.

### Ethics statement

This study was approved by the Institutional Review Board (IRB), Scientific Research Center, Prince Sultan Military Medical City, Riyadh, Saudi Arabia (IRB approval number 1657). Written informed consent was obtained from all participants. This study was conducted in accordance with the principles of the World Medical Association Declaration of Helsinki.

## Results

### Demographic and clinical data of participants

A total of 181 patients met the inclusion criteria and were enrolled in this study. More than half of the participants were women (*n* = 103, 56.9%). The mean age of participants (SD) was 58.64 ± 11.23 years with 42% (*n* = 76) >60 years of age and 35.4% (*n* = 64) 51–60 years of age. The mean duration of diabetes mellitus (SD) was 13.80 ± 8.55 years with more than half of the sample at >10 years since their diabetes diagnosis. Patients were recruited from three centers. The demographic and clinical data of participants are presented in Table 1.

**Table 1.** Demographic and Clinical Data of the Participants

Variable	Results
Sex, *n* (%)	
Male	78 (34.1)
Female	103 (56.9)
Age group, *n* (%)	
Mean ± SD	58.64 ± 11.23
≤40	7 (3.9)
41–50	34 (18.8)
51–60	64 (35.4)
>60	76 (42.0)
Duration of diabetes, *n* (%)	
Mean ± SD	13.80 ± 8.55
≤5	31 (17.1)
5–10	53 (29.3)
>10	97 (53.6)
Baseline HbA1c, mean ± SD	10.47 ± 1.23
BMI, mean ± SD	31.16 ± 6.43
Comorbidities, *n* (%)	
Hypertension	64 (35.4)
Dyslipidemia	95 (52.5)
Other comorbidities (CVD, CKD, hypothyroidism)	19 (10.50)
Primary center, *n* (%)	
Yasmine Center (A)	63 (34.8)
Almanar Center (B)	44 (24.3)
South Riyadh Center (C)	74 (40.9)

BMI, body mass index; CKD, chronic kidney disease; CVD, cardiovascular diseases; HbA1C, hemoglobin A1C; SD, standard deviation.

### Insulin therapy and dosage adjustment

#### Patients receiving insulin therapy

In this study, 79.6% of the patients (*n* = 144) were on insulin therapy, whereas 37 (20.4%) were not receiving insulin therapy. Of the patients receiving insulin therapy, 79 (54.9%) received one type of insulin, and 65 (45.1%) received two types of insulin. As shown in Table 2, of the 144 patients receiving insulin therapy, the majority (*n* = 136; 94.4%) were receiving Lantus either alone or with Aspart.

**Table 2.** Patients Receiving Insulin Therapy and Types of Insulin (*n* = 144)

Variable	***n*** (%)
Patients on insulin	
One type of insulin	79 (54.9)
Two types of insulin	65 (45.1)
Types of insulin^a^	
Novomix 30	6 (4.2)
Mixtard 70/30	2 (1.4)
Lantus^b^	136 (94.4)
Aspart^c^	65 (45.1)

^a^ Percentage is higher than 100% because some patients were on more than one type of insulin (total number of insulins = 209).

^b^ Out of the 136 patients on Lantus, 73 were on Lantus only, while 63 patients were on Lantus with Aspart.

^c^ Out of the 65 patients on Aspart, 63 were on Aspart with Lantus, while 2 were on Aspart with novomix 30.

#### Insulin dose adjustment during the telemedicine service

During tele-follow-up, the majority of patients on insulin therapy (*n* = 113; 78.5%) was provided with individualized guidance regarding dose adjustment by diabetes educators. These interventions were dose titration up in most patients and titration down in some patients to ensure appropriate insulin therapy according to the patient's clinical condition and glycemic targets (such as fasting blood glucose [FBG] and post-prandial blood glucose [PPBG]).

### Oral antidiabetic pharmacotherapy and non-insulin injectable medications

#### Types of oral and non-insulin injectable medications

The mean number of medications (SD) that the patients were prescribed was 2.1 ± 1 (median = 2, Q1–Q3 = 1–3). As shown in Table 3, metformin (*n* = 169; 88.45%), vildagliptin (*n* = 99; 54.7%), and gliclazide (*n* = 95; 52.2%) were the most commonly used medications.

**Table 3.** Types of Oral and Non-Insulin Injectable Medications

Variable	***n*** (%)^a^
Type of medication	
Metformin	160 (88.4)
Gliclazide	95 (52.2)
Vildagliptin	99 (54.7)
Semaglutide^b^	10 (5.5)
Empagliflozin	8 (4.4)
Linagliptin	4 (2.2)

^a^ Percentage is higher than 100% because some patients were on more than one medication (total number of medications = 376).

^b^ Injectable medication.

#### Interventions for medications

A total of 34 interventions have been made in consultation with the treating physician for non-insulin therapy. This represented a rate of 9.04% (34 interventions for the total number of prescribed medications, which was equal to 376). Of these interventions, nine were related to metformin (five increased the dose, three decreased the dose, and one discontinued the medication). Eleven interventions were related to gliclazide (four increased the dose, two decreased the dose, four discontinued the medication, and one added on). For semaglutide, there were six interventions (five increased the dose and one added on). There were two interventions (add-on) for linagliptin and two interventions for empagliflozin (one increased the dose and one added on). There were four interventions for vildagliptin (three discontinued the medication and one decreased the dose).

### Impact of the intervention on glycemic control

**FIG. 2.**
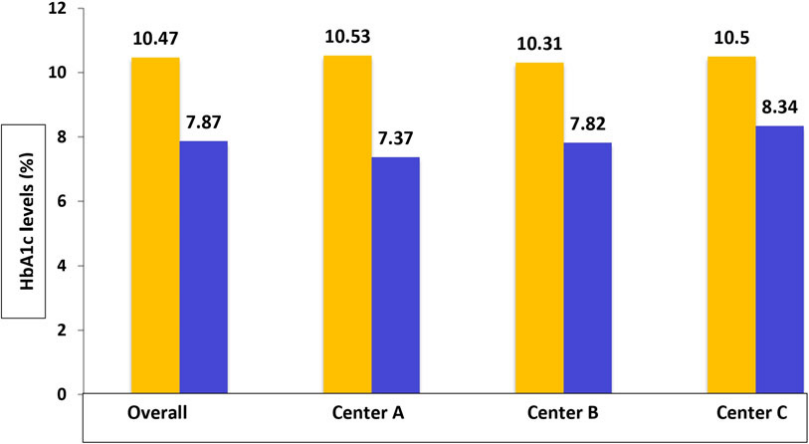
HbA1c levels (%) at baseline and end of the follow-up period. HbA1c, hemoglobin A1c.

The overall impact (*n* = 181; all three centers) and the impact at each center are presented in Table 4 and Figure 2. Overall, the intervention had significantly reduced the HbA1c from 10.47 ± 1.23% at pre-intervention to 7.87 ± 1.59% (*p* < 0.001). The intervention significantly reduced HbA1c levels at all three centers.

**Table 4.** The Overall and Center-Level Impacts of the Intervention on Glycemic Control

	Pre-intervention HbA1c	Post-intervention HbA1c	Mean difference (95% CI)	***p***
Overall	10.47 ± 1.23	7.87 ± 1.59	2.59 ± 1.73 (2.34–2.85)	<0.001
Center A	10.53 ± 1.10	7.37 ± 1.20	3.17 ± 1.43 (2.81–3.53)	<0.001
Center B	10.31 ± 1.29	7.82 ± 1.51	2.49 ± 1.87 (1.92–3.06)	<0.001
Center C	10.50 ± 1.31	8.34 ± 1.79	2.16 ± 1.75 (1.76–2.57)	<0.001

CI, confidence interval; HbA1C, hemoglobin A1C.

### Subgroup analysis

Subgroup analyses were conducted in terms of sex, age, duration of diabetes, insulin therapy, and oral pharmacotherapy. As shown in Table 5, the intervention significantly reduced HbA1c levels in all subgroups.

**Table 5.** The Subgroup Analyses of the Impact of Intervention on Hemoglobin A1C

Variable	Pre-intervention HbA1c	Post-intervention HbA1c	Mean difference (95% CI)	Intragroup difference ***p***-value
Sex				
Male	10.50 ± 1.20	7.98 ± 1.67	2.52 ± 1.86 (2.10–2.93)	<0.001
Female	10.44 ± 1.27	7.79 ± 1.51	2.65 ± 1.63 (2.33–2.97)	<0.001
Intergroup difference (*p* value)	0.755	0.419		
Age group				
≤40	10.27 ± 0.97	7.43 ± 1.08	2.84 ± 1.76 (1.22–4.47)	0.005
41–50	10.40 ± 1.05	8.07 ± 1.41	2.34 ± 1.68 (1.75–2.92)	<0.001
51–60	10.45 ± 1.22	7.76 ± 1.48	2.69 ± 1.64 (2.28–3.10)	<0.001
>60	10.53 ± 1.36	7.92 ± 1.79	2.69 ± 1.83 (2.19–3.02)	<0.001
Intergroup difference (*p* value)	0.932	0.699		
Duration of diabetes				
≤5	10.49 ± 1.19	7.73 ± 1.67	2.77 ± 1.76 (2.12–3.41)	<0.001
5–10	10.53 ± 1.29	7.62 ± 1.57	2.29 ± 1.95 (2.38–3.45)	<0.001
>10	10.42 ± 1.23	8.06 ± 1.56	2.36 ± 1.56 (2.04–2.67)	<0.001
Intergroup difference (*p* value)	0.856	0.227		
Insulin therapy				
No insulin therapy	10.14 ± 1.13	7.14 ± 0.95^*^^#^	2.99 ± 1.22 (2.59–3.41)	<0.001
One type of insulin therapy	10.46 ± 1.24	7.88 ± 1.49^*^	2.57 ± 1.91 (2.15–3.00)	<0.001
Two types of insulin therapy	10.66 ± 1.26	8.28 ± 1.83^#^	2.38 ± 1.72 (1.96–2.81)	<0.001
Intergroup difference (*p* value)	0.119	0.002		
Comorbidities				
No comorbidities	10.60 ± 1.23	7.75 ± 1.54	2.85 ± 1.58 (2.45–3.25)	<0.001
1 Comorbidity	10.40 ± 1.34	7.84 ± 1.67	2.55 ± 1.81 (2.12–2.99)	<0.001
≥2 comorbidities	10.39 ± 1.10	8.06 ± 1.54	2.33 ± 1.77 (1.83–2.82)	<0.001
Intergroup difference (*p* value)	0.551	0.579		

Symbols (^#^,^*^) indicate a statistically significant difference between the groups.

## Discussion

It is evident from the literature that achieving optimal glycemic control is challenging because a considerable proportion of patients have poorly controlled diabetes mellitus.^52,53^ This is despite the scientific, clinical, and technological advancements in antidiabetic therapies and diabetes management. As mentioned earlier, one of the key barriers to achieving optimal outcomes is therapeutic inertia, which is the failure to start or intensify insulin and antidiabetic therapy according to evidence-based guidelines.^54^

This is caused by several factors, including loss to follow-up, insufficient clinical consultation time, limited number of follow-up appointments, inadequate education provided to patients to allow starting or intensifying insulin therapy, anxiety about injections, fear of self-monitoring, perceived lack of self-efficacy, and inadequate training to perform self-care management.^55–58^ This further worsened during the COVID-19 pandemic, especially during its early phases.^39,59–62^ Consequently, one of the solutions to address these challenges is to implement innovative and flexible initiatives to educate and support patients, especially those receiving insulin and injectable therapy, and to coach patients during the initiation, intensification, and titration of insulin. This could be achieved by incorporating tele-education and frequent tele-follow-ups to ensure appropriate adherence to therapy and provide tailored guidance based on the patient's clinical condition and SMBG readings.

Consequently, we implemented a virtual diabetes education clinic as part of the primary diabetes care. In this model, frequent tele-follow-ups were conducted with patients, and relevant guidance and support were provided remotely to this high-risk group of patients.

The intervention reduced HbA1C substantially from a mean of 10.47 ± 1.23% (pre-intervention) to 7.87 ± 1.59% (post-intervention). These findings regarding the positive influence of virtual diabetes education, coaching, and clinical support on improving glycemic control in patients with T2DM are in line with those of earlier reports.^63–71^ Dixon et al. reported the impact of a virtual diabetes clinic (VDC) telehealth model for patients with T2DM, which aimed to provide support for the management of patients in primary care settings between office visits. VDC involves a mobile application with remote lifestyle coaching through CDEs and clinical support. The study reported statistically significant reductions in HbA1c by 2.3 ± 1.9% for patients with HbA1c >9%.^63^

A recent study in Thailand showed that diabetes self-management education and support delivered through telehealth are not inferior to in-person programs. At the 6-month follow-up, the reductions in HbA1c in the tele-health program and the in-person program were 1.28 ± 0.16% and 1.18 ± 0.15%, respectively.^70^ A recent review reported that telemedicine counseling was more effective than conventional counseling in reducing HbA1c levels in patients with diabetes in five of nine studies included in the review.^68^ Similarly, a scoping review of studies assessing the impact of telemedicine on self-care processes and therapeutic outcomes in patients with diabetes in the United States reported positive outcomes in terms of glycemic control, adherence to medications, blood glucose monitoring, and other self-care practices.^67^

Our study findings, in addition to recent scientific literature, suggest that virtual diabetes education, clinical support, and telemedicine services are viable solutions for effectively managing T2DM. Telemedicine and telecare are increasingly used to address inertia, provide health care support to patients with T2DM, ease accessibility to care, emphasize self-management behaviors, and improve clinical and quality of life outcomes.^72^ In addition, in our study, we noted that post-intervention, the level of HbA1c was relatively higher among those patients receiving multiple insulin therapies compared to those on less complex regimens. This indicates that this subgroup of patients might require additional attention to ensure their glycemic level is better controlled.

This study has several implications. This hybrid model of initial in-person (on-site) education, followed by individualized education, clinical support, and frequent follow-ups through telemedicine, was effective in managing patients with uncontrolled diabetes mellitus. Several factors have contributed to this achievement. First, the program was established with a clear setup, written operational procedures, and clear protocols and algorithms to form a clinical pathway that serves the purpose of good coordination among multidisciplinary health care professionals (treating physicians, diabetes educators, and clinical pharmacists).

Second, the patients in the initial in-person (on-site) visit to the diabetes education clinic were introduced to the program, its objectives, and the role of this service in helping them manage their disease and improve their quality of life. Third, during the on-site visit, it was an opportunity to train the patients who were newly prescribed insulin or their caregivers on the injection site and technique and to ensure that all patients were appropriately aware of the injection site, technique, and other related skills (such as the use of a glucometer and SMBG). Fourth, one of the key characteristics of this program is the frequent tele-follow-ups with patients to provide guidance and support, with emphasis on the safe and appropriate titration of insulin dose.

Diabetes educators helped the patients in a timely manner (weekly) with the adjustment of the diabetes care plan, especially insulin doses, starting with the recommended dose and titrating the dose upward or downward (in units) weekly according to the patient's glycemic targets until the desired glucose level was attained. This support was instrumental in helping patients effectively manage their diabetes between the physicians' office appointments. This type of program has the capacity to effectively tackle barriers such as therapeutic inertia by expediting informed treatment adjustments to prevent interruptions in the management of diabetes mellitus. Moreover, virtual interaction will ease and enhance patients' access to coaching programs, such as diabetes education.

Other benefits of the VDC, from the patients' perspective are that it could save time and be more convenient, especially with the frequency of visits, transportation, work leave, patients living in areas distant from the clinics, and associated costs; it could reduce the expenses related to chronic illness for patients and their caregivers owing to regular in-person clinic visits. In addition, it reduces the incidence of exposure to COVID-19 and other hospital-acquired infections by minimizing the number of nonessential hospital visits and face-to-face interactions. Overall, all these factors improve the control of diabetes mellitus and lead to overall satisfaction, which is linked with treatment compliance and improved quality of life.^73^

This study has several strengths. We believe that this is one of the few studies in the literature that has used a hybrid (combined) model of in-person and telemedicine education/follow-up for the management of high-risk T2DM patients. In addition, this was a prospective multi-center study with patients from three centers in different geographic locations in Riyadh city. Moreover, the effectiveness of the program was evaluated using the objective clinical measure of HbA1c. However, this study has some limitations. This study had a pre-post (before-and-after) design and did not involve another comparator group.

Moreover, the study did not assess long-term outcomes as this was not possible for the duration of the study. However, we believe that the findings of this study are valuable and provide further guidance to health policymakers when considering the implementation of telemedicine services and technologies to provide effective and convenient health care services to patients with diabetes. In addition, this study provides further evidence regarding the role of telemedicine in the self-management and support of patients with chronic diseases such as diabetes. This could provide better clinical outcomes in terms of glycemic control and improve the quality of life of patients.

## Conclusion

The findings showed that the hybrid model of in-person and telemedicine care and education was effective in managing uncontrolled T2DM during the COVID-19 pandemic. Consequently, the role of telemedicine in diabetes management can be further expanded as part of routine diabetes care in primary settings to achieve better glycemic control. In addition, the burden of nonessential in-person visits, especially frequent routine follow-ups, could be minimized and replaced with telemedicine care when appropriate.

## Notes

**Cite this article as:** Tourkmani AM, Alharbi TJ, BinRsheed AM, Alotaibi AF, Aleissa MS, Alotaibi S, Almutairi AS, Thomson J, Alshahrani AS, Alroyli HS, Almutairi HM, Aladwani MA, Alsheheri ER, Sati HS, Aljuaid B, Algarzai AS, Alabood A, Bushnag RA, Ghabban W, Albaik M, Aldahan S, Redda D, Almalki A, Almousa N, Aljehani M, Alrasheedy AA (2024) A hybrid model of in-person and telemedicine diabetes education and care for management of patients with uncontrolled type 2 diabetes mellitus: findings and implications from a multicenter prospective study, *Telemedicine Reports* 5:1, 46–57, DOI: 10.1089/tmr.2024.0003.

## Abbreviations Used

BMI: body mass index
CDEs: certified diabetes educators
CI: confidence interval
CICs: chronic illness centers
CKD: chronic kidney disease
CVD: cardiovascular diseases
HbA1C: hemoglobin A1C
IRB: Institutional Review Board
SD: standard deviation
SMBG: self-monitoring of blood glucose
T2DM: type 2 diabetes mellitus
VDC: virtual diabetes clinic
